# Atomic Layer Deposition of ScF_3_ and Sc_*x*_Al_*y*_F_*z*_ Thin Films

**DOI:** 10.1021/acsomega.3c09147

**Published:** 2024-02-26

**Authors:** Elisa Atosuo, Mikko J. Heikkilä, Johanna Majlund, Leevi Pesonen, Miia Mäntymäki, Kenichiro Mizohata, Markku Leskelä, Mikko Ritala

**Affiliations:** † Department of Chemistry, University of Helsinki, Helsinki 00014, Finland; ‡ Department of Physics, University of Helsinki, Helsinki 00014, Finland

## Abstract

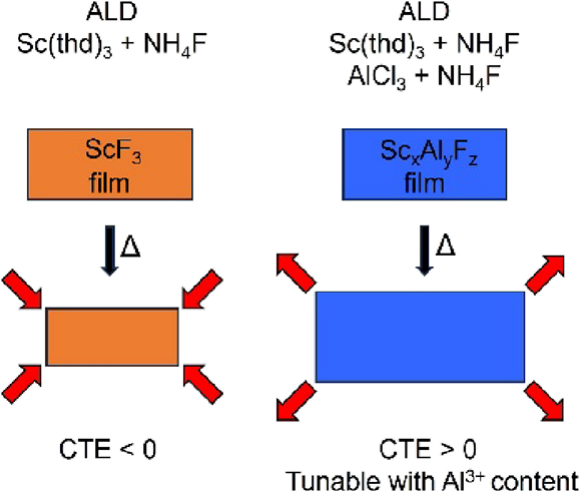

In this paper, we present an ALD process for ScF_3_ using Sc(thd)_3_ and NH_4_F as precursors. This is the first material made by ALD that has a negative thermal expansion over a wide-temperature range. Crystalline films were obtained at the deposition temperatures of 250–375 °C, with a growth per cycle (GPC) increasing along the deposition temperature from 0.16 to 0.23 Å. Saturation of the GPC with respect to precursor pulses and purges was studied at 300 °C. Saturation was achieved with Sc(thd)_3_, whereas soft saturation was achieved with NH_4_F. The thickness of the films grows linearly with the number of applied ALD cycles. The F/Sc ratio is 2.9:3.1 as measured by ToF-ERDA. The main impurity is hydrogen with a maximum content of 3.0 at %. Also carbon and oxygen impurities were found in the films with maximum contents of 0.5 and 1.6 at %. The ScF_3_ process was also combined with an ALD AlF_3_ process to deposit Sc_*x*_Al_*y*_F_*z*_ films. In the AlF_3_ process, AlCl_3_ and NH_4_F were used as precursors. It was possible to modify the thermal expansion properties of ScF_3_ by Al^3+^ addition. The ScF_3_ films shrink upon annealing, whereas the Sc_*x*_Al_*y*_F_*z*_ films show thermal expansion, as measured with HTXRD. The thermal expansion becomes more pronounced as the Al content in the film is increased.

## Introduction

Scandium is the lightest element in the series of rare earth metals. Contrary to many lanthanides, however, scandium does not have applications in the field of luminescence due to lack of 4f–4f transitions. Instead, an interesting property of scandium is the negative thermal expansion coefficient of scandium fluoride, encountered over a wide temperature range, approximately from 10 to 1100 K.^1^

Materials showing negative thermal expansion can counteract the thermal expansion of other materials in delicate applications, such as optical devices and semiconductor components. The counteracting role of ScF_3_ has been studied for example in Cu/ScF_3_ core–shell structures, where the motivation was to provide integrated circuit heat sinks with a similar thermal expansion coefficient to silicon.^2^ ScF_3_ has also been modified by adding Y^3+^, Ti^3+^, Al^3+^, Fe^3+^, Zr^4+^, and Fe^3+^ + Ga^3+^ ions into the structure.^3−8^ By adjusting the ion concentration, thermal expansion coefficients close to zero have been achieved.^5−8^

To the best of our knowledge, ScF_3_ has not yet been deposited by atomic layer deposition (ALD). ALD is an advanced thin film deposition method that is based on self-limiting reactions of alternately supplied gaseous precursors. The assets of ALD over many other thin film deposition methods are the stoichiometric, uniform, and conformal films it produces with excellent thickness control and reproducibility.^9^ Due to these properties, ALD is suitable for uniformly coating, e.g., nanoparticles or introducing dopants into films with uniform distribution. Thus, ALD is an excellent method for producing ScF_3_-based negative or zero thermal expansion materials.

In this article, an atomic layer deposition (ALD) process is presented for ScF_3_ using Sc(thd)_3_ and NH_4_F as precursors. Upon evaporation, NH_4_F decomposes to HF and NH_3_. The use of NH_4_F in ALD was first presented by Ylilammi and Ranta-aho^10^ By using NH_4_F, which is a solid precursor, the handling of commonly used but toxic HF is avoided. Also the metal impurity incorporation that is often observed when using metal fluoride precursors, such as TiF_4_, is avoided.

The ScF_3_ process presented here is the first ALD process for a wide-temperature-range negative thermal expansion (NTE) material. The process was also combined with an AlF_3_ ALD process to deposit Sc_*x*_Al_*y*_F_*z*_.

## Methods

All of the depositions were done with an F120 cross-flow reactor (ASM Microchemistry Oy). 99.999% nitrogen was used as the carrier and purging gas, and the depositions were done without further gas purification. The operating pressure of the reactor was 10 mbar. Sc(thd)_3_ (2,2,6,6-tetramethyl-3,5-heptanedionato scandium) was purchased from Volatec Oy, AlCl_3_ (99%) from Acros Organics and NH_4_F (≥99.99) from Sigma-Aldrich. All precursors were delivered from glass boats inside the ALD reactor and pulsed with an inert gas valving. The evaporation temperatures for Sc(thd)_3_, AlCl_3_ and NH_4_F were 105, 80, and 70 °C, respectively. The depositions were performed on Si substrates with the native oxide.

The thickness and refractive index of the films were measured by a Film Sense FS-1 Multiwavelength ellipsometer. Cauchy model was used for fitting. X-ray diffraction was measured with a PANalytical X’pert Pro MPD diffractometer, and the diffractograms were analyzed with PANalytical HighScore Plus software (version 4.7). High temperature XRD (HTXRD) was measured in a nitrogen atmosphere with the same diffractometer connected to an Anton Paar HTK1200N furnace. The 99.999% nitrogen was further purified with a MicroTorr MC1–902F gas purifier. The data were Rietveld refined with a MAUD 2.992 software.^11^

A Hitachi S-4800 field-emission scanning electron microscope (FESEM) was used for the morphology investigation. The same instrument connected to the Oxford INCA 350 microanalysis system was used for qualitative and quantitative energy dispersive X-ray spectroscopy (EDS) measurements. The Sc/Al ratios were determined from the EDS data using ScKα and AlKα lines with GMRFilm software. To increase the conductivity of the samples, a Au/Pd or carbon coating was applied prior to the FESEM and EDS measurements. Morphology of some of the films was further measured with a Veeco Multimode V atomic force microscopy (AFM) instrument. AFM measurements were done in tapping mode, and images were captured in air by using silicon probes with a nominal tip radius of 10 nm and a nominal spring constant of 5 N/m (Tap150 from Bruker). Images were flattened to remove artifacts caused by scanner bow and sample tilt. Roughness was calculated as a root-mean-square value (*R*_q_). The elemental composition was quantitatively determined with time-of-flight elastic recoil detection analyses (ToF-ERDA) using a 5 MV EGP-10-II tandem accelerator. 40 MeV ^79^Br^7+^ ions were used as bombarding ions with a detection angle of 40°.

## Results and Discussion

### ScF_3_ Films

The ScF_3_ deposition was first studied at deposition temperatures of 250–375 °C using 1 s pulse and purge lengths for both precursors. Film growth was observed at all temperatures, but the films deposited at 325 °C and higher had poor adhesion to the substrate and were partly flaking off. The effect was more pronounced when the deposition temperature was increased. According to the literature, Sc(thd)_3_ should be stable against decomposition up to at least 375 °C.^12^

**Figure 1:**
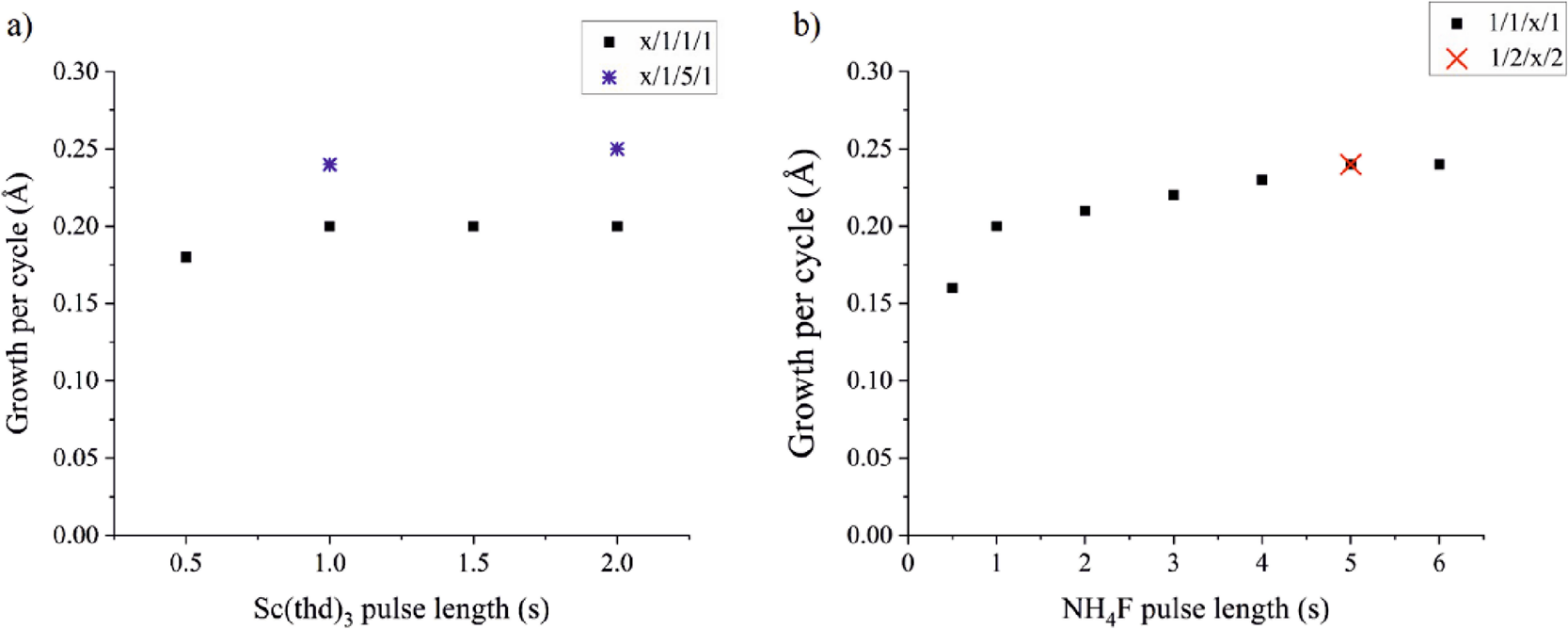
Growth per cycle as a function of (a) Sc(thd)_3_ pulse length and (b) NH_4_F pulse length.

Considering the flaking of the samples at high deposition temperatures, and on the other hand, the fact that the film purity usually increases as the deposition temperature is increased, the deposition temperature of 300 °C was chosen for the saturation tests. Figure 1a (black squares) depicts the growth per cycle (GPC) as a function of the Sc(thd)_3_ pulse length. The NH_4_F pulses and purges were 1 s. The GPC saturates to ∼0.2 Å/cycle with 1 s Sc(thd)_3_ pulses. The effect of the NH_4_F pulse length on the GPC was studied while the Sc(thd)_3_ pulses and purges were kept at 1 s (Figure 1b, black squares). The GPC saturates slowly toward 0.24 Å with 5.0 s of NH_4_F pulses. The same GPC is obtained also when both purges are 2 s, indicating that a 1 s purge time is sufficient (Figure 1b, red cross). Because the NH_4_F pulse length was not sufficient in the Sc(thd)_3_ pulse length studies, pulse lengths of 1 and 2 s were studied for Sc(thd)_3_ while keeping the NH_4_F pulses at 5 s (Figure 1a, blue stars). Only a slight increase is seen in the GPC, and 1 s Sc(thd)_3_ pulses can be considered sufficient.

**Figure 2:**
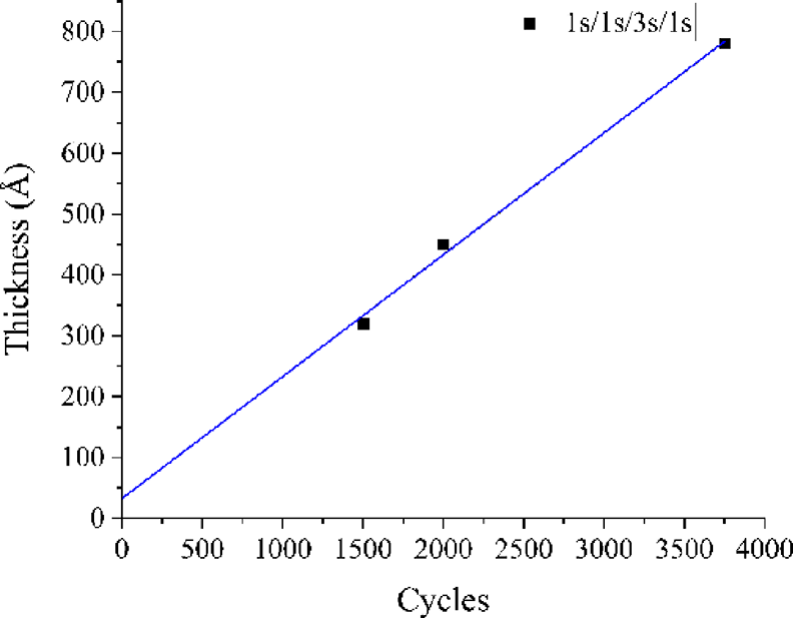
Thickness of the ScF_3_ films as a function of the applied ALD cycles.

The thicknesses of the films as a function of the number of ALD cycles was studied at a deposition temperature of 300 °C. Even when using a pulsing sequence of 1 s/1 s/3 s/1 s, which is slightly unsaturated with respect to the NH_4_F pulses, the thickness is linearly dependent on the applied ScF_3_ cycles (Figure 2).

**Figure 3:**
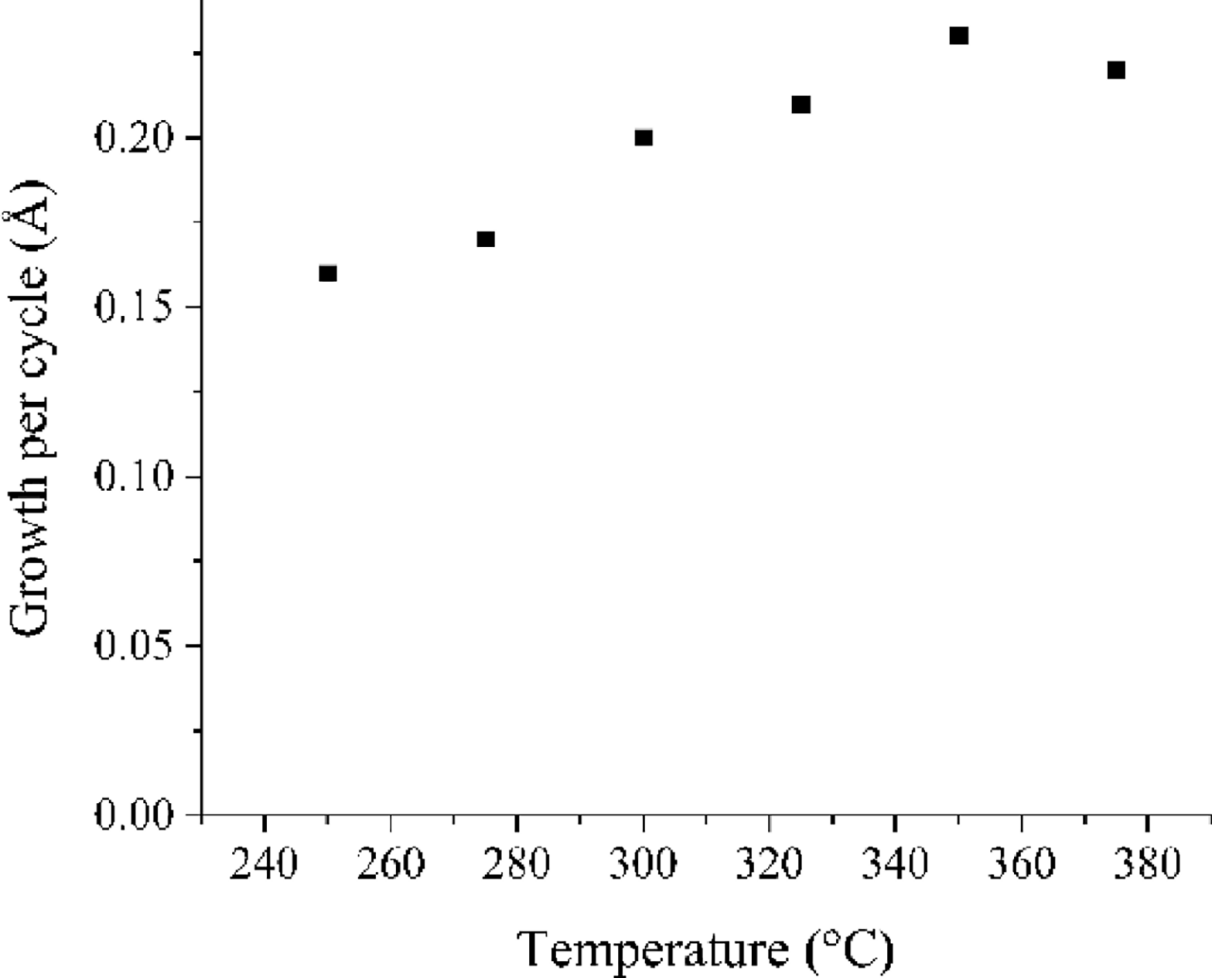
GPC as a function of the deposition temperature.

The GPC values as a function of the deposition temperature are depicted in Figure 3 for films deposited with 1500 cycles. Similar to LiF and GdF_3_ ALD processes using NH_4_F as the fluorine source, the GPC increases as a function of the deposition temperature.^13,14^ The GPC is similar to the GdF_3_ ALD process where the GPC was 0.22–0.26 Å.^14^

**Figure 4:**
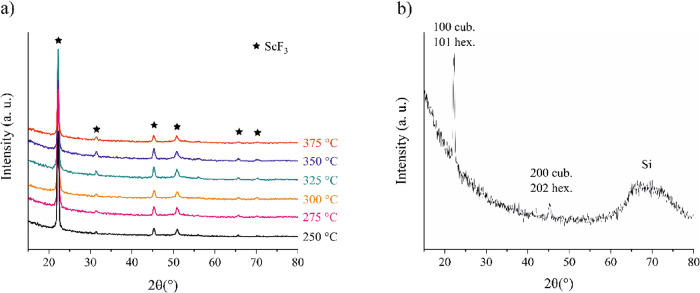
(a) GIXRD of films deposited at 250–375 °C and (b) 2θ-ω measurement (offset 4°) of a film deposited at 300 °C.

Figure 4a shows the grazing incidence X-ray diffractograms (GIXRD) of ∼25–35 nm films deposited at 250–375 °C. All the deposition temperatures resulted in crystalline films. The diffraction patterns of the cubic, hexagonal, and rhombohedral ScF_3_ overlap, complicating the analysis. The film deposited at 300 °C was measured also in the 2θ-ω mode with 4° offset, which means that only the planes nearly parallel to the film surface are probed and Si substrate peaks are avoided. As seen in Figure 4b, the diffractogram obtained with the 2θ-ω scan is not similar to the reference pattern, indicating a preferred orientation. Thus, the relative intensities of the GIXRD reflections can not be used to identify the phase.

**Figure 5:**
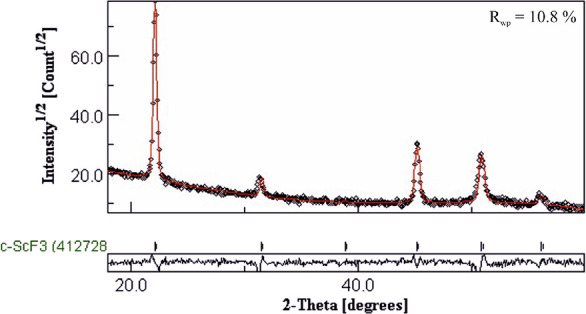
Rietveld fit of the XRD data of the ScF_3_ film. The black circles present the measured data, whereas the red line presents the fitting. The plot below shows the deviation of the fitting from the data.

The GIXRD data of the film deposited at 300 °C were Rietveld refined using the MAUD software package. Cubic, hexagonal, and rhombohedral crystal forms were attempted, and the best fit to the data was achieved using the cubic form. However, some of the reflections are shifted with respect to the reference patterns, which is an indication that the phase is either strained cubic ScF_3_ or contains an additional phase. The films were therefore annealed to see whether some relaxation would occur. When kept at 300 °C for 4 h in nitrogen atmosphere, shifts in the positions of some of the reflections occurred improving the fit to the cubic phase though still not matching perfectly. Figure 5 shows the diffractogram, the Rietveld fit, and the quality of the fit (*R*_wp_ value) of the annealed film.

The composition and stoichiometry of the films deposited at 250–350 °C were determined with ToF-ERDA (Table 1). The F/Sc ratio ranges from 2.9 to 3.1 as expected from the XRD results. The hydrogen content is moderate, max. 3.0 at %, whereas the carbon and oxygen contents are low. Only the carbon content is clearly temperature dependent: the higher the deposition temperature the lower the carbon content. No nitrogen was found in the films, despite NH_3_ forming upon NH_4_F decomposition.

**Table 1:** Stoichiometry and Impurity Contents of ScF_3_ Films Deposited at 250–350 °C as Measured by ToF-ERDA

*T*_dep_ (°C)	Sc	F	O	N	C	H	stoichiometry
250	23.6 ± 0.4	72.0 ± 0.9	1.64 ± 0.10		0.49 ± 0.06	2.3 ± 0.5	3.1
275	24.2 ± 0.5	71.0 ± 1.0	1.33 ± 0.09		0.40 ± 0.06	3.0 ± 0.7	2.9
300	23.9 ± 0.4	73.5 ± 0.9	1.26 ± 0.08		0.23 ± 0.04	1.2 ± 0.3	3.1
325	23.7 ± 0.4	72.7 ± 0.8	1.31 ± 0.09		0.15 ± 0.02	2.2 ± 0.5	3.1
350	23.9 ± 0.5	73.0 ± 0.9	1.22 ± 0.08		0.12 ± 0.02	1.8 ± 0.4	3.1

As in previous studies on ALD metal fluoride films, also the ScF_3_ films eroded fast during the ToF-ERDA measurements.^14,15^ Interestingly, there was a clear trend in the erosion rates of the measured ScF_3_ films. The fastest erosion was observed with the film deposited at the lowest temperature and the slowest with the film deposited at the highest temperature. The reasons for this remain unknown and might be related to morphology or roughness. The stoichiometries and impurity contents are similar and are therefore not likely to affect the erosion rates. The densities of the films were measured by XRR from films deposited at 300 and 325 °C. The density of these films is the same, 2.6 g/cm^3^, and therefore, density is not a probable reason for the different behavior.

**Figure 6:**
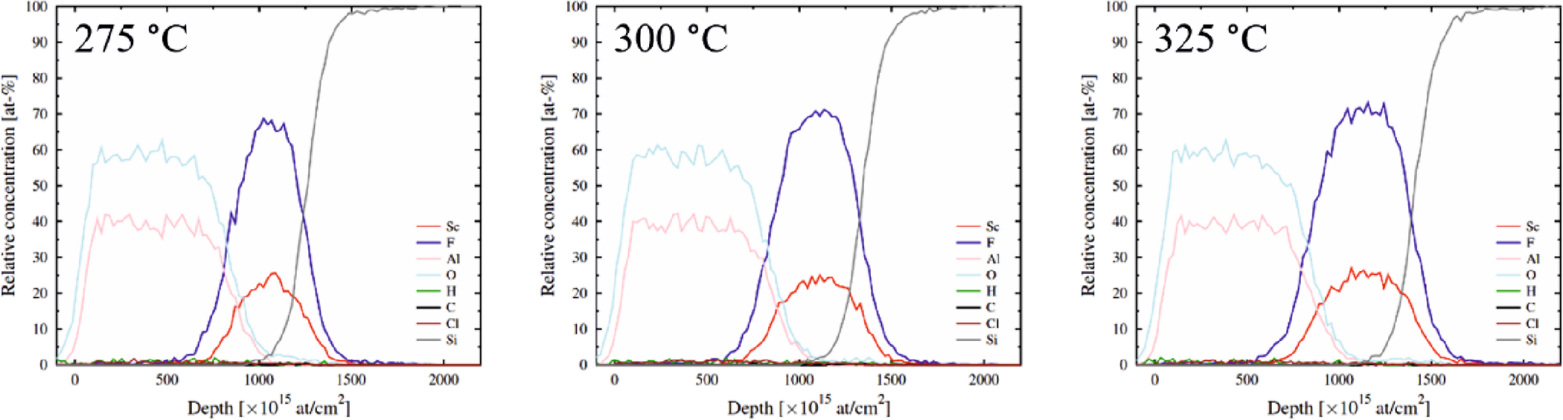
Depth profiles of *ex situ* alumina capped ScF_3_ films deposited at 275 (∼65 nm), 300 (∼80 nm), and 325 °C (∼80 nm).

The ToF-ERDA depth profiles are not reliable due to fast erosion. However, it is possible to prevent the erosion to some extent by using a capping layer on top of the films.^14^Figure 6 shows depth profiles of ScF_3_ films deposited at 275, 300, and 325 °C and capped with ALD-alumina after breaking the vacuum. The capping was performed at 300 °C using the AlCl_3_ + H_2_O process. As seen, the depth distributions of scandium and fluoride match well.

**Figure 7:**
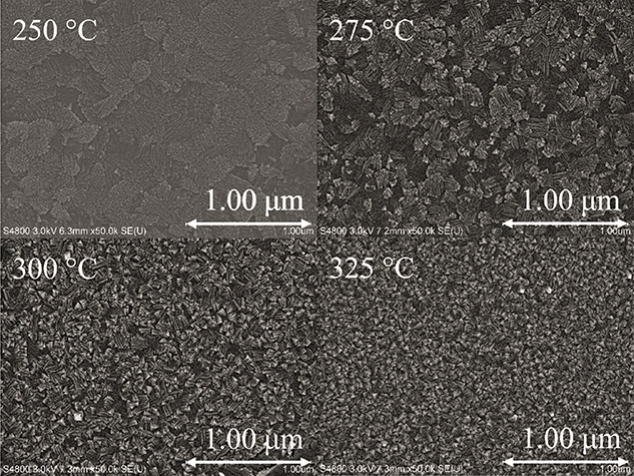
FESEM images of films deposited at 250–325 °C. The thicknesses of the films are 71, 63, 78, and 85 nm for 250, 275, 300, and 325 °C, respectively.

Figure 7 shows field-emission scanning electron microscope (FESEM) images of ∼60–85 nm thick films deposited at 250–325 °C. The films contain lamellar-like grains (Figure 7), which is visible especially in the sample deposited at 275 °C. Similar lamellar structures have been observed in other ALD metal fluorides, such as yttrium fluoride and holmium fluoride.^16,17^

**Figure 8:**
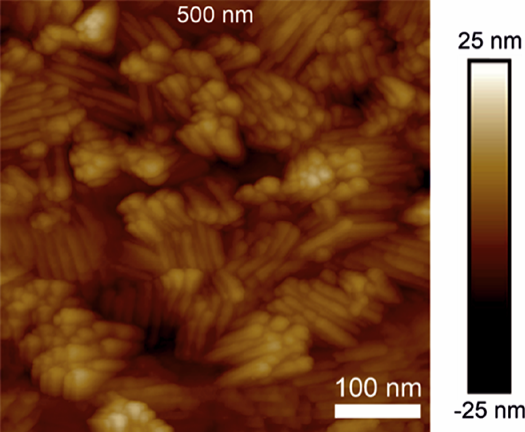
AFM image of a ScF_3_ film deposited at 275 °C.

**Figure 9:**
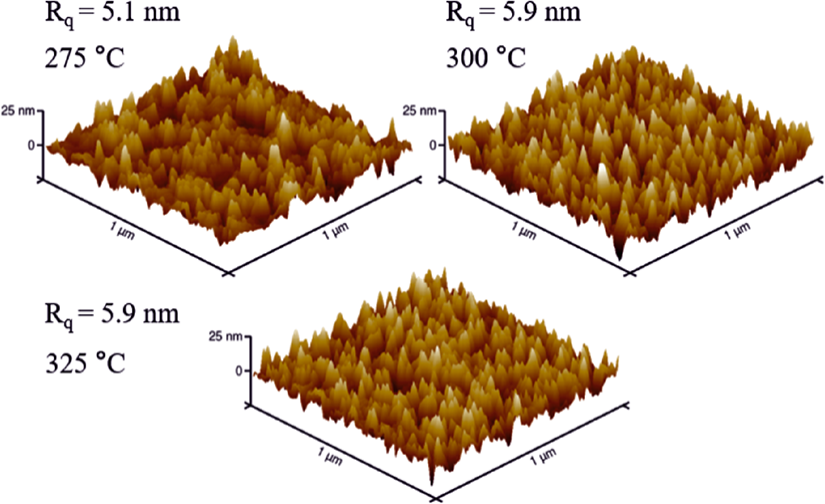
AFM images of films deposited at 275–325 °C. The thicknesses of the films are 63, 78, and 85 nm for 275, 300, and 325 °C, respectively.

The same films were measured with an atomic force microscope (AFM). The lamellar structure is seen also in AFM as shown in Figure 8 for the film deposited at 275 °C. The root-mean-square roughness (*R*_q_) values are similar in all the films, 5.1 nm for the film deposited at 275 °C and 5.9 nm for the films deposited at 300 and 325 °C (Figure 9). Since the film deposited at the lowest temperature was the thinnest and the film deposited at the highest temperature was the thickest, the roughness seems to decrease with the increasing deposition temperature.

The thermal expansion of the ScF_3_ films was studied by HTXRD in a nitrogen atmosphere. The film was preheated for 4 h at 300 °C to relax any internal film stress, as explained earlier. The HTXRD measurements were done in the temperature range of 25–345 °C, because according to preliminary tests, the films oxidize at ∼425 °C and one even at 345 °C. This occurred even though the furnace was preheated at 300 °C in nitrogen atmosphere for 1 h while simultaneously pumping with a turbomolecular pump to remove any moisture remaining in the porous ceramic insulation material.

**Figure 10:**
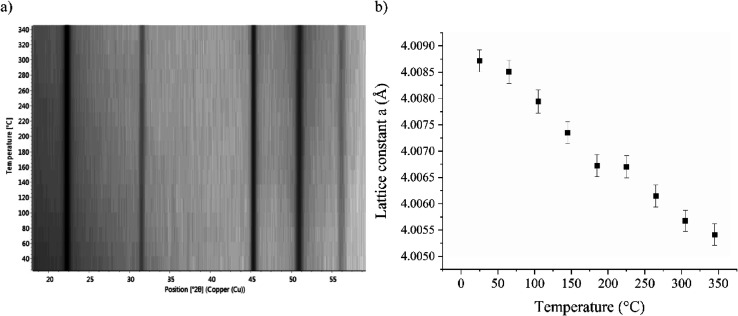
(a) HTXRD of a preheated ScF_3_ film and (b) temperature dependence of the lattice parameter *a* of the film as modeled using the cubic ScF_3_ model.

**Figure 11:**
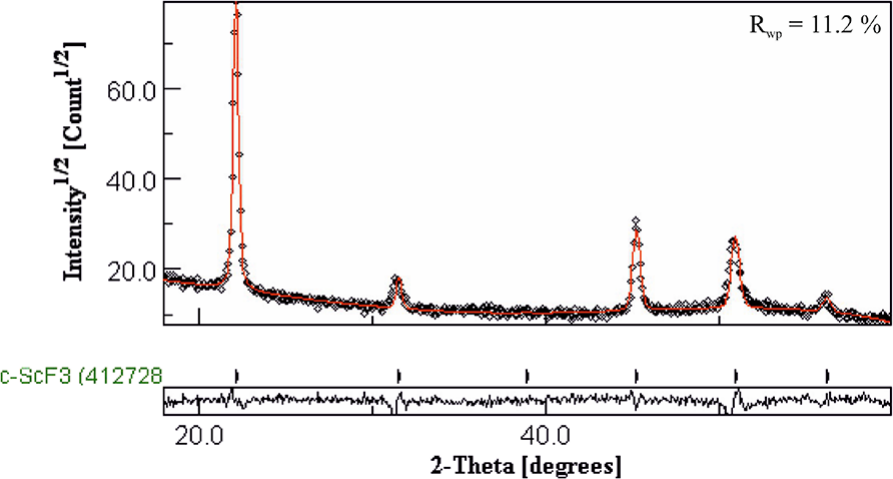
Rietveld fit of the XRD data of the ScF_3_ film measured at 185 °C. The black circles present the measured data, whereas the red line presents the fitting. The plot below shows the deviation of the fitting from the data.

Figure 10a shows the HTXRD measurements of a 94 nm thick film. The positions of the reflections do not change much. The unit cell parameter was determined at each temperature with the MAUD software (Figure 10b). The lattice constant at room temperature is 4.0087 Å, and it decreases to 4.0054 Å as the temperature is increased, indicating negative thermal expansion, i.e., contraction. The diffractogram, the Rietveld fit and the *R*_wp_ value at 185 °C are shown in Figure 11.

To our knowledge, there are no previous reports on the thermal expansion properties of ScF_3_ in thin film form. In this work, the unit cell parameter versus temperature data were fitted with a second order polynomial: *a*(*T*) *= a*_0_ + *a*_1_*T + a*_2_*T*^2^, where *a*_0_, *a*_1_ and *a*_2_ are the fitting parameters. From the definition of the linear thermal expansion coefficient
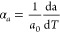
we obtain the thermal dependence of the lattice constant *a* as
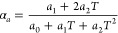


As an example, the linear thermal expansion coefficients of an undoped ScF_3_ film are −2.4 × 10^–6^ K^–1^ at 25 °C and −2.3 × 10^–6^ K^–1^ at 52 °C.

In the bulk form, ScF_3_ is reported to have a linear thermal expansion coefficient α = −3.88 × 10^–6^ K^–1^ in the temperature range of 325–675 K (52–402 °C).^18^ Thermal properties of thin films can differ from the bulk, for example, due to stress caused by the mismatch between the film and the substrate. In addition, the thickness of the film might affect the thermal properties. ScF_3_ particle size has been shown to affect the thermal expansion coefficient.^19^

### Sc_*x*_Al_*y*_F_*z*_ Films

From the application point of view, the main aim in studying negative thermal expansion materials is to obtain a zero thermal expansion material. In the literature, these kinds of materials have been obtained for example by the addition of Al^3+^ ions in ScF_3_ powder.^5^ Our aim was to investigate whether Al^3+^ ions could be incorporated in the ScF_3_ films by combining the ScF_3_ ALD process with an AlF_3_ ALD process.

The original idea was to combine ScF_3_ cycles with Al(thd)_3_ + NH_4_F cycles in a supercycle manner. However, the precursor combination of Al(thd)_3_ + NH_4_F did not result in film growth. Earlier it has been shown by Mäntymäki et al. that the combination of Al(thd)_3_ and TiF_4_ does not produce any film on Si, and poor-quality films were grown on LiF.^20^ Therefore, the combination of AlCl_3_ and NH_4_F was chosen to be studied. To our knowledge, the combination of AlCl_3_ and NH_4_F has not been used in AlF_3_ ALD processes before. In literature, AlF_3_ has been deposited by using the precursor combinations of AlCl_3_ + TiF_4_, AlMe_3_ + TaF_5_, AlMe_3_ + HF/pyridine, and Al(NMe_2_)_3_ + HF.^20−24^

**Figure 12:**
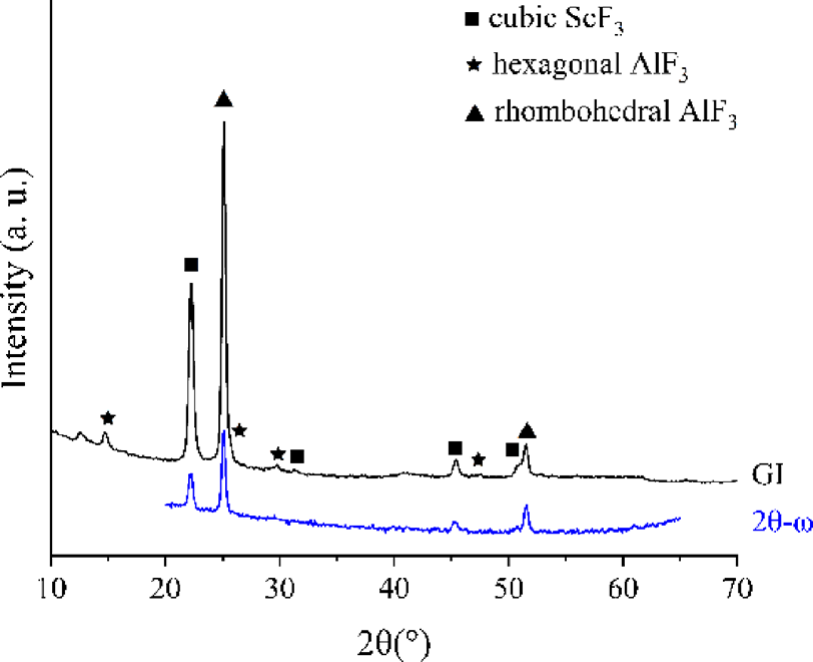
GIXRD and 2θ-ω measurements on an AlF_3_ film deposited on ScF_3_.

The growth was studied at a deposition temperature of 300 °C on both Si and ScF_3_ surfaces. The ScF_3_ films were approximately 30 nm thick and were deposited at 300 °C. The GPC was ∼0.3 Å on Si and ∼1.3 Å on ScF_3_ according to ellipsometry. According to GIXRD, the AlF_3_ films grow amorphous on silicon but are crystalline on ScF_3_ substrates. The film on ScF_3_ contains both hexagonal (ICDD PDF 43-435) and rhombohedral (ICDD 44-231) AlF_3_ (Figure 12). There is an additional unknown reflection present, however. A 2θ-ω scan with a 4° offset was measured to investigate whether the films were orientated. As seen in Figure 12, the rhombohedral phase is orientated whereas the hexagonal phase is hardly seen, indicating that it may be located on the surface.

**Figure 13:**
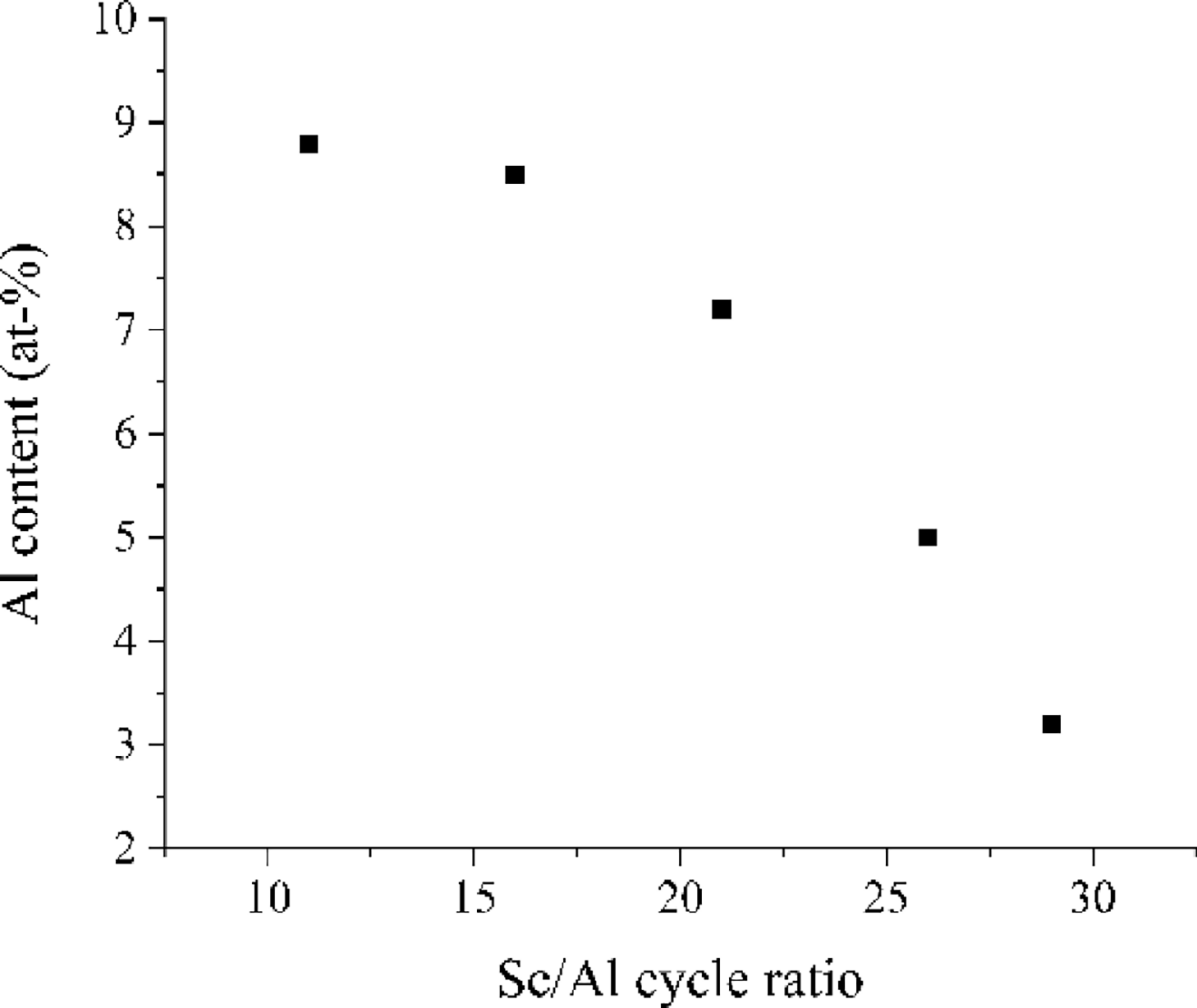
Al content in atomic percents as measured by EDS as a function of the Sc/Al binary cycle ratio.

Sc_*x*_Al_*y*_F_*z*_ films were deposited at 300 °C by varying the ratio of the binary cycles of ScF_3_ and AlF_3_, e.g., 1000(10(Sc(thd)_3_ + NH_4_F) + 1(AlCl_3_ + NH_4_F)). In the AlF_3_ binary cycle, a 1 s/1.5 s/3 s/3 s pulsing sequence was used. The Al content was studied with EDS by comparing the Sc and Al atomic percentages (Figure 13).

**Figure 14:**
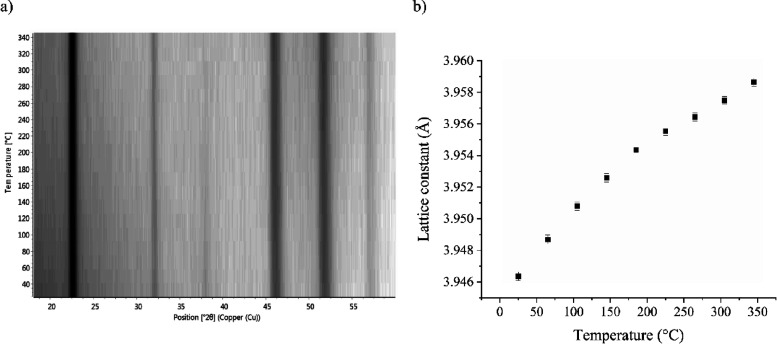
(a) HTXRD of a preheated Sc_*x*_Al_*y*_F_*z*_ film and (b) temperature dependence of the lattice parameter *a* of the film as modeled using the cubic ScF_3_ model.

The X-ray diffractograms of the Sc_*x*_Al_*y*_F_*z*_ films are otherwise similar to those of the ScF_3_ film, but there is an additional unidentified reflection at the 2θ position of ∼38°. A 116 nm thick film with the Al content of 4.3 at % (Sc/Al ratio 0.83:0.17) was measured with HTXRD after preheating it at 300 °C for 4 h (Figure 14a). The film with the Sc/Al ratio of 0.83:0.17 was chosen, because in the literature close to zero thermal expansion material was obtained when the Sc/Al ratio of a powder was 0.85:0.15.^5^

**Figure 15:**
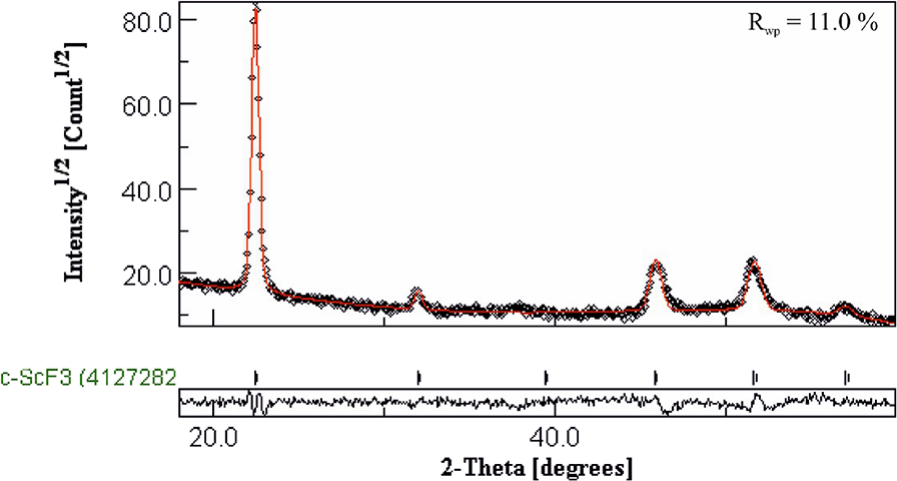
Rietveld fit of the XRD data of the Sc_*x*_Al_*y*_F_*z*_ film measured at 185 °C. The black circles present the measured data, whereas the red line presents the fitting. The plot below shows the deviation of the fitting from the data.

The intensity of the unidentified reflection decreases upon annealing but increases again upon cooling. This might indicate that the additional reflection belongs to rhombohedral Sc_*x*_Al_*y*_F_*z*_ which changes to cubic Sc_*x*_Al_*y*_F_*z*_ as has been reported in literature.^5^ In general, the positions of the other reflections shift to lower angles, indicating that the film expands during annealing (Figure 14a). Attempts were made to fit the data with either cubic or rhombohedral ScF_3_ or a combination of both. The data was best fitted with the cubic ScF_3_, and the modeled unit cell parameters are shown in Figure 14b. For example, the lattice parameter at room temperature is 3.9464 Å, and it increases to 3.9586 Å as the temperature is increased to 345 °C. The Rietveld fit of the data at 185 °C is shown as an example (Figure 15).

**Figure 16:**
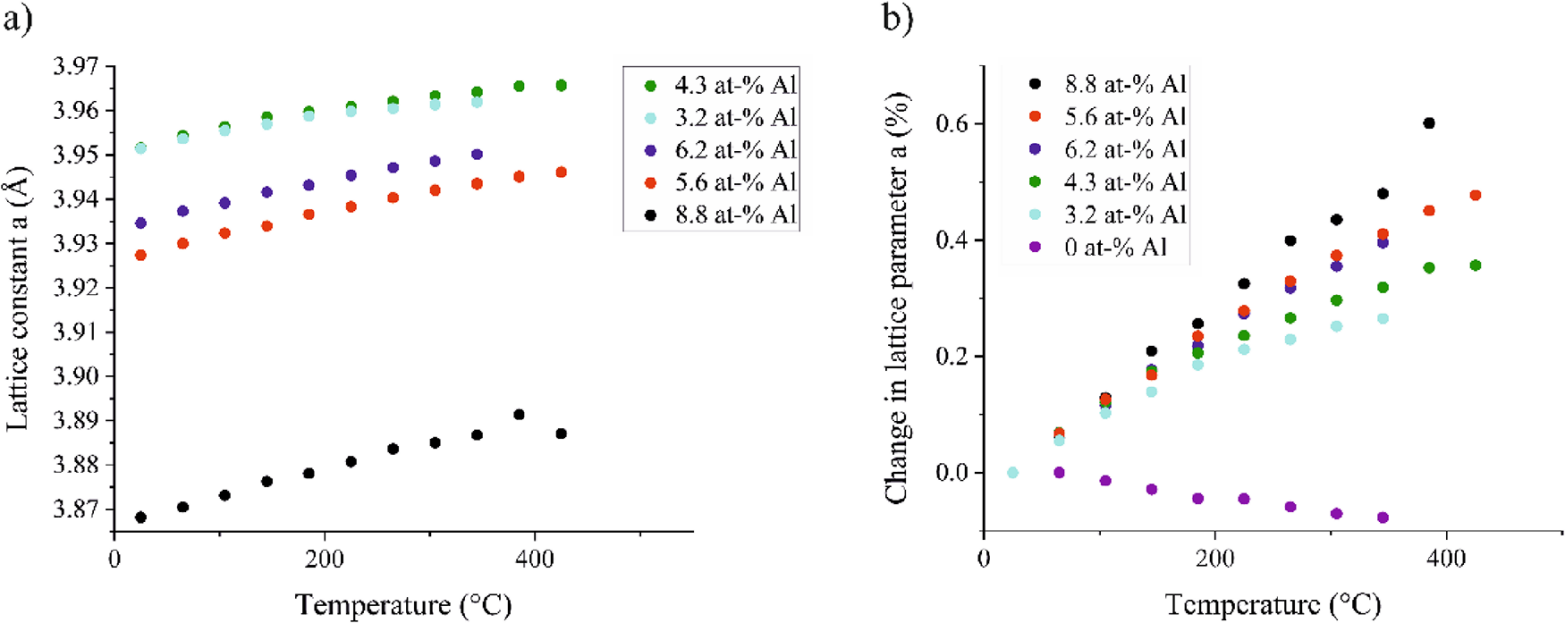
(a) Lattice constants of films with different Sc/Al ratios as a function of temperature and (b) the change (%) in the lattice constant as compared to room temperature lattice constant in ScF_3_ film and films with different Sc/Al ratios.

Similar HTXRD results were obtained when the film was not preheated. Films with different Sc:Al ratios were therefore measured with HTXRD without preheating. Figure 16a presents the modeled lattice parameters as a function of the temperature for these films. The lattice constant of the film with the largest Al content (Sc/Al ratio 0.65:0.35 and total Al content 8.8 at %) is much smaller than that of the other films. The film with the smallest Al contents 4.3 and 3.2 at % (0.83:0.17 and 0.87:0.13) have the largest lattice constants and they are very close to each other. The third largest lattice constant is measured in a film with 6.2 at % Al content (0.75:0.25) and in close proximity is the film with 5.6 at % Al content (0.78:0.22). The lattice constants of the films with Al contents of 5.6 and 6.2 at % are thus in reverse order than what was expected. This might be explained by the uncertainty in the Sc/Al ratios. The much smaller lattice constant in the film with the largest Al content might in turn be explained by the residual stress in the film.

Figure 16b shows the percentual change in the lattice constant as compared to the room temperature lattice constant in films with different Sc/Al ratios. As expected, the percentual change in the lattice constant is the largest in the film with the largest Al content, approximately 0.48% at 345 °C. The films with Al contents of 5.6 and 6.2 at % have very similar values with each other, 0.41 and 0.40%. As expected, the lowest percentual change 0.27% is in the film with the lowest Al content. The percent change in the lattice constant of the undoped ScF_3_ film is included in the figure for comparison.

The calculated linear thermal expansion coefficients at 25 °C range from 4.0 × 10^–6^ K^–1^ for the film with a 3.2 at % Al content to 10.3 × 10^–6^ K^–1^ for the film with an 8.8 at % Al content.

## Conclusions

An ALD process for ScF_3_ was studied by using Sc(thd)_3_ and NH_4_F as precursors. The films grow crystalline at the studied deposition temperatures of 250–375 °C. The GPC increases along the deposition temperature from 0.16 to 0.23 Å. The F/Sc ratio is 2.9–3.1 as measured by ToF-ERDA. Small hydrogen, carbon and oxygen contents were found in the films, and their maximum contents are 3.0, 0.5, and 1.6 at %, respectively. Nitrogen was not found in the films.

The saturation of the GPC with respect to precursor pulses and purges was studied at 300 °C. Saturation of the GPC was achieved with Sc(thd)_3_ pulses, but soft saturation was achieved with the NH_4_F pulses. Linear thickness increase with the number of applied ALD cycles was observed. The film morphology is lamellar type, especially at lower deposition temperatures, as investigated by FESEM. The films are rough; for example, for a 78 nm film deposited at 300 °C the *R*_q_ is 5.9 nm.

ScF_3_ films were also doped with Al^3+^ by combining Sc(thd)_3_ + NH_4_F and AlCl_3_ + NH_4_F binary ALD cycles at 300 °C. The ScF_3_ films were measured with HTXRD and showed a negative thermal expansion. In turn, the Sc_*x*_Al_*y*_F_*z*_ films expand upon annealing, and the expansion is more pronounced as the Al content increases. It is thus possible to tune the thermal expansion properties of ScF_3_ by the addition of Al^3+^ ions to the structure. It is assumed that by finding the right Sc/Al ratio, obtaining a zero thermal expansion material with ALD is possible.
